# Cross-Sectional and Quasi-Longitudinal Examination of Childhood and Adult Cognitive Disengagement Syndrome, Depression, Anxiety, Stress, and Insomnia

**DOI:** 10.3390/jcm14145165

**Published:** 2025-07-21

**Authors:** Dena Sadeghi-Bahmani, Larina Eisenhut, Thorsten Mikoteit, Nico Helfenstein, Annette Beatrix Brühl, Kenneth M. Dürsteler, Jean-Marie Bizimana, Stephen P. Becker, Serge Brand

**Affiliations:** 1 Department of Psychology, Stanford University, Stanford, CA 94305, USA; bahmanid@stanford.edu; 2 Center for Affective, Stress and Sleep Disorders, Psychiatric Hospital of the University of Basel, 4002 Basel, Switzerland; larina.eisenhut@unibas.ch (L.E.); thorsten.mikoteit@spital.so.ch (T.M.); annette.bruehl@upk.ch (A.B.B.); 3 Psychiatric Services of Solothurn, University of Basel, 4503 Solothurn, Switzerland; 4 Sport Science Section, Department of Sport and Health Science, Faculty of Medicine, University of Basel, 4002 Basel, Switzerland; nico.helfenstein@stud.unibas.ch; 5 Division of Substance Use Disorders, Psychiatric University Clinics, 4002 Basel, Switzerland; kenneth.duersteler@upk.ch; 6 Department for Psychiatry, Psychotherapy and Psychosomatic, Psychiatric Hospital, University of Zurich, 8091 Zurich, Switzerland; 7 Centre de Psychiatrie et Psychothérapie Les Toises, 1023 Lausanne, Switzerland; jean-marie.bizimana@lestoises.ch; 8 Division of Behavioral Medicine and Clinical Psychology, Cincinnati Children’s Hospital Medical Center, Cincinnati, OH 45229, USA; stephen.becker@cchmc.org; 9 Department of Pediatrics, University of Cincinnati College of Medicine, Cincinnati, OH 45267, USA; 10 Health Institute, Substance Abuse Prevention Research Center, Department of Psychiatry, Kermanshah University of Medical Sciences (KUMS), Kermanshah 6714686698, Iran; 11 Sleep Disorders Research Center, Department of Psychiatry, Kermanshah University of Medical Sciences (KUMS), Kermanshah 6714686698, Iran; 12 School of Medicine, Tehran University of Medical Sciences, Tehran 1416753955, Iran; 13 Center for Disaster Psychiatry and Disaster Psychology, Center of Competence of Disaster Medicine of the Swiss Armed Forces, 4002 Basel, Switzerland

**Keywords:** cognitive disengagement syndrome, quasi-longitudinal study, childhood, adulthood, depression, anxiety, stress, insomnia, conditional effect models, sluggish cognitive tempo

## Abstract

**Background:** Longitudinal studies on cognitive disengagement syndrome (CDS) are scarce, and only one study has investigated the trajectory of CDS from childhood to early adulthood. Given this, the aims of the present study were to explore, with a quasi-longitudinal design, (1) whether scores for childhood CDS were associated with scores for CDS during early adulthood; (2) whether childhood CDS scores were associated with childhood and adult scores for depression, anxiety, stress, and insomnia; (3) whether childhood CDS, depression, anxiety, stress, and insomnia and adult depression, anxiety, stress, and insomnia were independently associated with adult scores for CDS, and (4) whether childhood CDS scores were directly and indirectly associated with adult CDS scores via adult depression and stress in two conditional effect models. **Methods:** A total of 246 young adult students (mean age = 22.62; 56.3% females) participated in a cross-sectional and quasi-longitudinal study. The participants completed questionnaires assessing CDS (Adult Concentration Inventory; ACI), depression, anxiety, stress, and insomnia for the following two developmental periods: for the present time point as young adults and for a past time point, when they were about eight years old. To enable retrospective past recall, the participants undertook a standardized imagination exercise. **Results:** Childhood scores for CDS, depression, anxiety, stress, and insomnia were highly associated with adult scores for CDS, depression, anxiety, stress, and insomnia. In the regression model, higher childhood scores for CDS, depression, and anxiety and higher adult scores for depression, stress, and insomnia, but not adult anxiety, were strongly and independently associated with adult scores for CDS. In the two conditional effects models, childhood CDS was associated with adult CDS directly and indirectly via adult depression and adult stress. **Conclusions:** In this quasi-longitudinal study, childhood scores for CDS were associated with adult scores for CDS, suggesting a potentially stable trajectory of CDS from childhood to early adulthood. Further, the two conditional effects models suggested that childhood and adult CDS were both directly and indirectly associated via adult depression and stress. As such, symptoms of depression, anxiety, stress, and insomnia should be considered in conceptualizations of adult CDS. Next, given that standardized psychotherapeutic interventions for depression, stress, and insomnia are available, such interventions might also favorably impact CDS symptoms. These findings further underscore the importance of prospective longitudinal and intervention studies on adult CDS.

## 1. Introduction

Over the last two decades, both the scientific community and experts working in clinical settings have paid increasing attention to cognitive disengagement syndrome (CDS; formerly sluggish cognitive tempo) among children, adolescents, and adults [1,2]. CDS refers to a set of behaviors including daydreaming, staring, mental fogginess/confusion, slowed behavior/thinking, and hypoactivity [1,3]. More specifically, CDS comprises both cognitive disengagement (e.g., in a fog/confused and stares/preoccupied/in own world) and hypoactivity (e.g., sluggish/slow moving/low energy, drowsy/sleepy/not alert, and tires easily) [4,5]. Importantly, along with a recent consensus report and overview on CDS [1,2], meta-analytic results have repeatedly shown that CDS is also factor-analytically distinguished from the inattentive symptoms of attention-deficit/hyperactivity disorder (ADHD-IN; [6,7]) and from autism spectrum disorder [5]. More specifically, CDS appears to have an internal source of distraction, whereas ADHD-IN may be more clearly related to external distraction [8]. This would also explain why individuals with CDS do not necessarily appear distracted and why they still appear focused when completing tasks. The slower cognitive processing implicated also provides evidence that CDS is different from ADHD in general and ADHD-IN specifically [7]. Next, whereas ADHD is strongly associated with disruptive behavior and externalizing symptoms, CDS is more strongly associated with internalizing symptoms such as depression and anxiety [2,9,10], and also potentially with sleep disturbances [10,11,12,13,14,15]. Last, while the number of cross-sectional CDS studies among children, adolescents, and adults continues to increase [2], few longitudinal studies focused on CDS have been conducted. We identified the following 14 longitudinal studies. 

### 1.1. Longitudinal Studies on CDS During Childhood

The test–retest reliability of parent-rated CDS scores among children aged approximately seven years old remained stable over a time span of four to six weeks (rs = 0.79–0.80; [16]), while the test–retest reliability (rs = 0.61–0.74) of teachers’ ratings of 758 first graders (about 8 years old) also remained fairly stable over one year for the following items: seems drowsy, thinking slow, and slow moving [17]. For the same sample, parent-rated scores remained stable (rs = 0.46–0.42) over two years for the following items: seems drowsy, slow moving, thinking slow, loses train of thought, and easily confused [18]; see also [19].

Becker et al. [20] assessed processing speed and executive functions among 1002 five-year-old children and teacher-rated scores for SCT/CDS and ADHD-IN three to five years later, when the children were in grades one to three. A lower processing speed at baseline predicted greater SCT/CDS and ADHD-IN behavior at follow-up. Higher inhibitory control and higher working memory abilities predicted lower ADHD-IN scores, but not SCT/CDS behavior. 

### 1.2. Longitudinal Studies on CDS from Childhood to Adolescence

Mayes et al. [21] reported data on 376 young people (mean age at baseline: 8.7 years, and mean age at follow-up: 16.4 years). CDS behaviors were assessed using the caregiver-completed Pediatric Behavior Scale, and the key results were as follows: 1. Higher scores for CDS at baseline during childhood were the strongest predictor for higher CDS scores in adolescence. 2. Higher scores for autism spectrum disorder and insomnia at baseline predicted higher scores for CDS in adolescence, independently of CDS scores at baseline. 3. Higher scores for autism, insomnia, inattention, somatic complaints, and excessive sleep were related to CDS at baseline and at follow-up. 4. Higher scores for depression at follow-up were associated with higher scores for CDS at the same time point. 5. Baseline hyperactivity/impulsivity was negatively associated with baseline CDS. Mayes et al. [21] concluded that CDS during childhood was the strongest risk factor for CDS during adolescence, followed by higher scores for autism and insomnia. 

Darow et al. [22] assessed 68 children with spina bifida (SB; mean age: 8.34 years) and 68 typically developing peers (TD; mean age: 8.49 years) both at baseline and eight years later. Growth curves indicated that CDS scores steadily increased over time in both groups. Further, higher levels of teacher-reported (but not mother-reported) CDS at baseline predicted worse social skills for young people both with and without SB in adolescence. Next, regarding the slope findings, higher rates of mother-reported CDS over time predicted worse social skills (β = −0.43) and lower levels of youth decision making (β = −0.43) for the SB group, while higher rates of teacher-reported CDS predicted worse social skills for the TD group. Overall, these data showed that scores for CDS increased over time in a sample of children crossing into adolescence with and without spina bifida. 

In a quasi-longitudinal and cross-sectional study, Burns et al. [23] assessed the parent-reported CDS scores of 5525 children and adolescents (56.1% boys); the age ranges were early childhood (ages 5–8), middle childhood (ages 9–12), and adolescence (ages 13–16). Among others, the results showed that CDS symptoms showed a convergent and discriminant validity relative to ADHD-IN symptoms and that CDS showed stronger first-order and unique associations than ADHD-IN with anxiety, depression, somatization, daytime sleep-related impairment, and nighttime sleep disturbance. Importantly, such patterns of results were observed within each age group, suggesting a strong pattern of CDS score stability from childhood to early and mid-adolescence. 

Leopold et al. [24] assessed 489 preschoolers at age five and then ten years later. The authors observed that scores for CDS and ADHD remained generally stable from childhood to mid-adolescence, though hyperactivity/impulsivity scores slightly decreased, inattention scores remained stable, and CDS scores slightly increased. 

Becker et al. [25] assessed 188 first to sixth graders (6–13 years; 47% boys; teacher ratings) and 133 nine-to-12-year-old children (child self-report ratings) both at baseline and six months later. The pattern of results was sophisticated: higher SCT/CDS scores at baseline predicted higher scores for depression (teacher ratings and child ratings) and anxiety (teacher ratings) and higher scores for anxiety, but not depression, predicted higher scores for SCT/CDS later on. The anxiety-SCT/CDS-link was informant-independent. 

Dvorsky et al. [26] assessed 1173 children aged between 3 and 12 years. The mean levels of SCT/CDS increased modestly with age and became more prominent between five and seven years, and lower parent education was associated with higher parent- and teacher-reported SCT/CDS. Overall, SCT/CDS could be observed as early as early childhood. 

By contrast, Vu et al. [27] reported that among 639 children aged from 6 to 12 years, there was a low longitudinal stability (r < 0.60) of SCT/CDS scores over a time span from one to two or from one to seven years. Further, higher SCT/CDS scores predicted greater social problems, internalizing behaviors, and anxious/depressive behavior, even when controlling for ADHD scores. By contrast, SCT/CDS scores had no significant independent effects on cognitive educational outcomes after the effects of ADHD-IN were controlled for.

### 1.3. Longitudinal Studies on CDS During Adolescence

Fredrick et al. [28] assessed 302 participants crossing into adolescence (mean age: 13.17 years; 44.7% females) two years later. Higher self-reported scores for CDS at baseline uniquely predicted higher scores for depression two years later. Importantly, higher scores for peer victimization mediated the association between higher CDS scores and higher depression scores two years later. 

### 1.4. Longitudinal Study from Childhood and Adolescence to (Early) Adulthood

Smith et al. [29] assessed 449 children and adolescents 12 years later. At baseline, children and adolescents completed self-rated questionnaires on CDS, depression, and ADHD. Higher scores for CDS and depression predicted higher scores for depression 12 years later, and higher scores for CDS predicted higher ADHD-IN scores. 

### 1.5. The Present Study

Studies on the trajectory of CDS are scarce, with only 14 studies identified; these studies assessed children over trajectories of some weeks [16], months [25], and up to seven years [17,18,19,20,27], as well as children crossing into adolescence (e.g., [21,22,23,24]), while one study assessed trajectories during adolescence [28] and one study investigated CDS trajectories from childhood/adolescence to early adulthood [29]. The general pattern was that scores for CDS remained stable [21,23] or even increased [22,24,26] over time.

To summarize, to our understanding, there is only one longitudinal study on CDS from childhood to adulthood. Given this, we performed a cross-sectional and quasi-longitudinal study on adult CDS. To methodologically justify the procedure, we identified the following five previous studies where quasi-longitudinal designs were successfully applied: 1. Trembath et al. [30] identified retrospectively assessed patterns of physical activity and lifestyle during childhood, adolescence, and early adulthood as predictors of the year of onset of Huntington’s Disease in adulthood. 2. Khazaie et al. [31] showed that current patterns of non-suicidal self-injury were associated with retrospectively assessed past non-suicidal self-injury. 3. Schmidt et al. [32] showed that adult females’ current physical activity patterns were associated with previous, retrospectively assessed, and remembered physical activity patterns. 4. Bader et al. [33,34] showed that retrospectively assessed and remembered parenting styles during childhood and adolescence were associated with adult insomnia, and most recently, 5. Sadeghi-Bahmani et al. [35] showed that retrospectively assessed and remembered prenatal stress was associated with an increased risk of developing post-partum depression 12 weeks after delivery. Given these previously successfully applied methodological procedures, we also applied the quasi-longitudinal design technique in the present study. 

Our two hypotheses and two research questions were as follows: First, following others [17,18,19,21,22,23,28,29], we predicted that scores for CDS would remain fairly stable over time. Second, following others [1,2,9,21,36], we assumed that higher scores for CDS at both time points (early adulthood and childhood) would be associated with higher scores for depression, anxiety, stress, and insomnia (‘psychological ill-being’).

The first research question was as follows: Which childhood and adulthood dimensions of psychological ill-being (depression, anxiety, stress, and insomnia), along with childhood CDS, are more strongly and independently associated with CDS in adulthood? To answer this question, we conducted a multiple regression model.

The second research question was as follows: Is childhood CDS associated directly or also indirectly with adult CDS via dimensions of psychological ill-being? To answer this question, we conducted two conditional effect models; such conditional effect models are allowed when cross-sectional and quasi-longitudinal data are treated as if they were assessed at two clearly distinguishable time points. In the latter case, mediation models would be the correct statistical approach [37,38].

## 2. Methods

### 2.1. Procedure

As already previously reported [9], students at the University of Basel (Basel, Switzerland) were invited to participate in an anonymous online study, which was performed with the online survey software Tivian^®^/Questback^®^. The manufacturer warrants that all data are securely stored on the manufacturer’s server, that no third parties have access to the data, and that no hidden participant information such as IP address will be gathered and stored.

On the first page of the online study (see also [9]), participants were fully informed about the aims of the study, the anonymous data gathering, and data elaboration. Participants were also informed that participation or non-participation had no advantages or disadvantages for the continuation of the study, and that they could stop or interrupt their participation at any time without any further justification. The first page of the online study further indicated that the present data might be used for scientific research and publication, that questionnaires were filled out for the present time as adults and for when they were children, and that audio instruction would be used to help them recall their experience during childhood. Next, to ‘sign’ the written informed consent, participants were asked to tick the following box: “I have understood the aims of the study, including the anonymous data gathering and the secure and anonymous data handling. I know that I can withdraw from the study without further consequences, and I know, who I contact in case of further study-related questions.” Afterwards, participants completed a booklet of self-rated questionnaires covering sociodemographic information, symptoms of CDS, and symptoms of psychological ill-being, that is, depression, anxiety, stress, and insomnia. For symptoms of CDS, depression, anxiety, stress, and insomnia during childhood, participants listened to audio based on imagination techniques (see below for details). On average, participants needed about 50–60 min to complete the online questionnaires (see details below). The study lasted from 11 November 2024 to 31 December 2024. The local ethical committee (Ethikkommission Nordwest- und Zentralschweiz [EKNZ], Basel, Switzerland) approved the study (Register-nr: Req-2024-01463; approved on 11 November 2024), which was performed according to the seventh [39] and current version of the Declaration of Helsinki. 

In a previous paper [9], we investigated the associations between adult CDS and psychological ill-being in this sample; one manuscript was accepted for publication with the core data set, where we investigated if and to what extent adult CDS scores were associated with patterns of physical activity, and in a further submitted manuscript, we investigated the associations between adolescent and adult CDS; however, the data reported in the present manuscript are novel; thus, there is a low risk of publication overlap. 

### 2.2. Participants

The inclusion criteria were as follows: 1. aged between 18 and 30 years; 2. student at the University of Basel (Basel, Switzerland); 3. willing and able to comply with the study requirements, including an intermediate to mastery level in German; and 4. ticking the box to “sign” the written informed consent. The exclusion criteria were as follows: 1. withdrawing from participation; 2. pregnancy or breastfeeding, as such stages may alter both mood and sleep; and 3. ‘click-throughs’ who needed less than five minutes to complete the questionnaires.

A total of 746 individuals read the first page, 512 (68.63%) started the online survey, and 246 (32.98%) completed it. Of those, one (0.13%) self-declared to be gender non-binary; given this very low prevalence rate of individuals self-declaring as non-binary, this data set was not further considered for data analysis. The full data set consisted of 245 participants (32.84%; mean age: 22.62 years (SD = 3.10; 56.3% female)).

### 2.3. Measures

#### 2.3.1. Sociodemographic Information

Participants reported their gender (male; female; and non-binary) and age (years). 

#### 2.3.2. Adult Concentration Inventory (ACI)

The ACI is a self-report measure to assess CDS symptoms [40,41,42]. Example items include “I’m slow at doing things”, “I get lost or drowsy during the day”, and “I get lost in my own thoughts”. Each item is rated on a four-point scale (0 = not at all, 1 = sometimes, 2 = often, and 3 = very often), with a higher sum score reflecting greater CDS symptom severity (Cronbach’s alpha: 0.94). As in previous studies [10,42], we used a 15-item version without the low motivation item, as several studies found the “I’m not motivated” item to cross-load or primarily load on an ADHD-IN factor rather than a CDS factor [43,44,45]. As mentioned elsewhere [9,42], we translated the questionnaire following standardized algorithms [46].

#### 2.3.3. Depression, Anxiety, Stress

To assess symptoms of depression, anxiety, and stress, participants completed the Depression, Anxiety, Stress-questionnaire (DASS-21; [47,48]). The questionnaire consists of 21 items, and example items include “I felt down and depressed” [depression]; “I felt I was close to panic” [anxiety]; and “I was in a state of nervous tension” [stress]. Answers are given on 4-point Likert scales ranging from 0 (=does not apply to me at all) to 3 (=extremely applies to me), with higher mean scores reflecting a greater severity of symptoms. Accordingly, the depression, anxiety, and stress subscales were calculated separately (Cronbach’s alpha of the overall score = 0.96).

#### 2.3.4. Insomnia

To assess insomnia, participants completed the German version [49] of the Insomnia Severity Index (ISI; [50]). It includes seven questions about sleep quality and insomnia, and participants answered how often certain issues concerning sleep quality had occurred during the last month on scales ranging from 0 (=never/not at all) to 4 (=always). The total score ranges from 0 to 28, with a higher sum score reflecting a greater severity of insomnia (Cronbach’s alpha: 0.88). The following cut-off values are proposed [50]: 0–7 points: no insomnia; 8–14 points: subthreshold insomnia: 15–21 points: moderately clinically relevant insomnia; and 22–28 points: severely clinically relevant insomnia.

### 2.4. Imagination Intervention to Recall the Past as a Child

Before completing the questionnaires on CDS, depression, anxiety, stress, and insomnia during childhood, to facilitate recall, participants listened to the following audio:

To recall the time you were a child, when you were eight years old, the present audio will help you.

Stay comfortably seated and close your eyes [pause of 5”]. My eyes are closed and I breathe calmly and regularly [repeated 5 times]. I’m at primary school; I see the building, I see my classmates; I feel the scent of the building and the classroom; I see my teachers and classmates. Now, I’m at home; I see the apartment where I’m living; I’m in my bedroom and lying on my bed; I see my room, my books, my bookshelf, the posters on the wall, the window, my closet, including my favorite sweaters and ts; I feel the scent of my bedroom. My eyes are closed and I breathe calmly and regularly [repeated 5 times]. I’m doing my home works; I remember my best friends, my favorite toys, my favorite movies, and my favorite teachers; I remember the summer break holidays. My eyes are closed and I breathe calmly and regularly [repeated 5 times]. I feel being fully back to my childhood. My eyes are closed and I breathe calmly and regularly [repeated 5 times]. I’m a child; I see my bedroom, my school peers, the school, my favorite sweater, and I’m playing with my favorite toys; I remember very well my birthday party, Christmas time with my family and relatives. Now, I’m coming back, and with the feeling of being a child, I complete the next questionnaires.

The imagination techniques were adapted to the current study aims from standardized CBT imagination techniques for insomnia [51] and for relaxation in the general population [52] and among professional athletes [53,54]. 

### 2.5. Analytic Plan

####  Preliminary Calculations

With a series of Pearson’s correlations, we investigated if age was systematically associated with childhood and adult CDS, depression, anxiety, stress, and insomnia. All rs values were <0.11 (ps > 0.35). Thus, age was not introduced as confounder. 

With a series of t-tests, we investigated if male and female participants systematically differed in age and child and adult CDS. Compared to males (M = 21.90; SD = 2.79), females were older (M = 23.19; SD = 3.22; t(243) = 3.30, *p* < 0.01, d = 0.43) and reported higher adult CDS scores (males: M = 8.91 (SD = 7.52); females: M = 11.40 (SD = 9.32; t(243= = 2.25, *p* < 0.05, d = 0.29)), but not statistically higher child CDS scores (males: M = 6.80 (SD = 8.74); females: M = 8.04 (SD = 9.67; t(243= = 1.04, *p* = 0.30, d = 0.13)). Given the small effect sizes, the decision was not to introduce sex as a confounder. 

With a series of Pearson’s correlations, we explored the associations between adult and child scores for CDS, depression, anxiety, stress, and insomnia. 

To explore whether adult scores for depression, anxiety, stress, and insomnia and childhood scores for CDS, depression, anxiety, stress, and insomnia were independently and more strongly associated with adult CDS scores, a multiple regression analysis was performed. The statistical requirements to run a multiple regression model were met [55,56,57], as follows: N = 245 > 100; predictors explained the dependent variable (R = 0.856; R^2^ = 0.734); number of predictors: 9 (adult depression, anxiety, stress, and insomnia; child CDS, depression, anxiety, depression, and insomnia): 10 × 9 = 90 < N (245); the Durbin–Watson coefficient was 1.98, indicating that the residuals of the predictors were independent. Last, the variance inflation factors (VIFs) were between 2.977 and 4.374; while there are no strict cut-off points to report the risk of multicollinearity, VIF < 1 and VIF > 10 indicate multicollinearity [55,56,57]. 

Last, given that mediating models derived from cross-sectional and quasi-longitudinal data lead to biased results [37,38], we ran two conditional effects models. 

The level of significance was set at alpha < 0.05. All statistical computations were performed with SPSS^®^ 29.00 (IBM Corporation, Armonk, NY, USA) for Apple Mac^®^ (Cupertino CA, USA). 

## 3. Results

### 3.1. General Information

Full data were available for 245 participants. Their mean age was 22.62 years (SD = 3.10); 107 (43.7%) were males, and 138 (56.3%) were females. 

### 3.2. Correlations Between Adulthood CDS, Depression, Anxiety, Stress, and Insomnia and Childhood CDS, Depression, Anxiety, and Stress

Table 1 reports the Pearson’s correlation coefficients, along with the descriptive statistical indices, for adulthood and childhood scores for CDS, depression, anxiety, depression, and insomnia.

Higher adulthood CDS scores were associated with higher adulthood depression, anxiety, stress, and insomnia scores. Likewise, higher adulthood CDS scores were associated with higher childhood CDS, depression, anxiety, stress, and insomnia scores.
Higher adulthood depression scores were associated with higher adulthood anxiety, stress, and insomnia scores and with higher childhood CDS, depression, anxiety, stress, and insomnia scores.
Higher adulthood anxiety scores were associated with higher adulthood stress and insomnia scores and with higher childhood CDS, depression, anxiety, stress, and insomnia scores.
Higher adulthood stress scores were associated with higher adulthood insomnia scores and with higher childhood CDS, depression, anxiety, stress, and insomnia scores.
Higher adulthood insomnia scores were associated with higher childhood CDS, depression, anxiety, stress, and insomnia scores.
Higher childhood CDS scores were associated with higher childhood depression, anxiety, stress, and insomnia scores.
Higher childhood depression scores were associated with higher childhood anxiety, stress, and insomnia scores.
Higher childhood anxiety scores were associated with higher childhood stress and insomnia scores.
Higher childhood stress scores were associated with higher childhood insomnia scores.

### 3.3. Regression Model to Identify Which Adult and Childhood Dimensions Were More Strongly Associated with Adult CDS Scores

To answer the research question regarding which adulthood and childhood dimensions were more strongly and independently associated with adulthood CDS, a multiple regression model was performed with adulthood CDS as dependent variable and adulthood depression, anxiety, stress, insomnia and childhood CDS, depression, anxiety, stress, and insomnia as independent variables (Table 2).

Higher childhood CDS scores, higher childhood depression and anxiety scores, and higher depression, stress, and insomnia scores were independently associated with higher adulthood CDS scores. Descriptively, childhood CDS scores had the highest beta value. Adulthood anxiety scores and childhood stress and insomnia did not reach statistical significance. 

### 3.4. Conditional Effects Models to Identify the Direct and Indirect Associations of Childhood CDS with Adult CDS via Adult Depression (Model 1) and via Adult Stress (Model 2)

**Figure 1:**
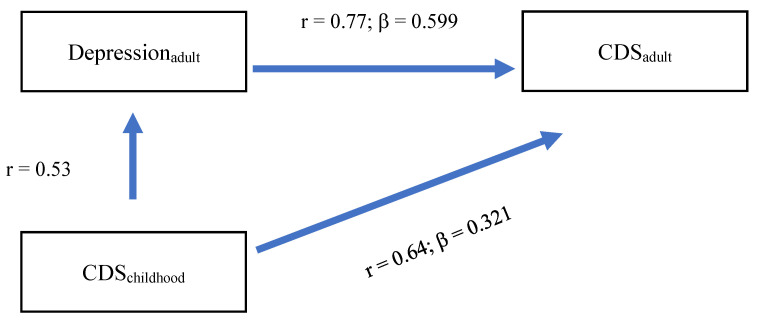
Model 1 Direct associations between childhood CDS and adulthood CDS, and indirect associations between childhood CDS and adulthood CDS via adulthood depression.

**Figure 2:**
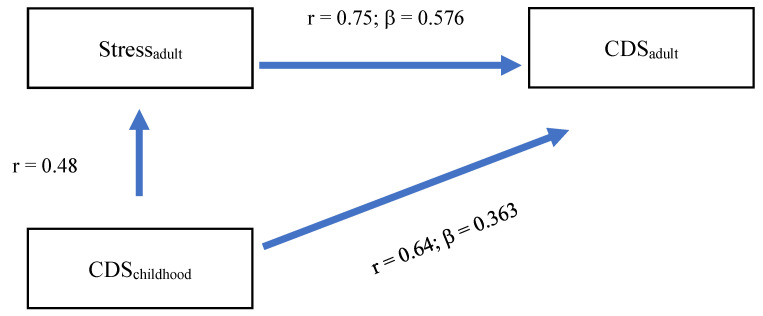
Model 2 Direct associations between childhood CDS and adulthood CDS, and indirect associations between childhood CDS and adulthood CDS via adult stress.

From the correlational computations (Table 1) and the regression model (Table 2), the question arose of whether childhood CDS scores were directly or indirectly associated with adult CDS scores via adult depression scores (Model 1; Figure 1) and whether childhood CDS scores were directly and indirectly associated with adult CDS scores via adult stress scores (Model 2; Figure 2). To test such conditional effects models, we followed Rudolf and Mueller [56], considering that betas may differ from the main regression model.

The equation model is as follows:

r_CDSchildhood-adult-_ = β_CDSchildhood_ + (r_CDchildhood-depression-adult_ × β_depression-CDSadult_)

0.638 = 0.321 + (0.53 × 0.599)

0.638 = 0.321 + 0.3175

Overall, the model showed that there was both a direct association of childhood CDS with adult CDS and an indirect association of childhood CDS with adult CDS via adult depression. 

The equation model is as follows:

r_CDSchildhood-adult-_ = β_CDSchildhood_ + (r_CDSchildhood-Stressaadult_ × β_stress)_

0.64 = 0.363 + (0.48 × 0.576)

0.64 = 0.363 + 0.220

Overall, the model showed that childhood CDS was associated with adult CDS both directly and indirectly via increased adult stress scores.

## 4. Discussion

The aims of the present cross-sectional and quasi-longitudinal study were to investigate if and to what extent scores for cognitive disengagement syndrome (CDS) in childhood were associated with CDS scores in early adulthood, along with childhood and adulthood psychological ill-being (depression, anxiety, stress, and insomnia). The results showed that (a) childhood and adulthood scores for CDS were highly associated; (b) childhood and adulthood scores for CDS, depression, anxiety, stress, and insomnia were associated; (c) childhood CDS, depression, and anxiety and adulthood depression, stress, and insomnia scores were independently and strongly associated with adulthood CDS; and (d) in two conditional effect models, childhood CDS scores were associated with adulthood CDS both directly and independently and indirectly via adulthood depression and stress. The present results add to the current literature on CDS in adulthood in the following three ways. First, the quasi-longitudinal design showed that childhood CDS remained stable and even slightly and descriptively increased in early adulthood; second, childhood and adulthood CDS scores were associated with psychological ill-being (depression, anxiety, stress, and insomnia) in childhood and adulthood; and third, childhood CDS was directly and indirectly associated with adulthood CDS via adulthood depression and stress, suggesting a strong CDS–depression–stress link both cross-sectionally and longitudinally.

The two hypotheses and two research questions formulated are considered now in turn.

### 4.1. Stability of CDS Scores over Time

Following others [17,18,19,21,22,23,28,29], we predicted that scores for CDS would remain fairly stable over time, and data did confirm this. Importantly, we also confirmed the previous observation that CDS may increase over time [24], in that mean CDS scores increased from 7.50 to 10.31 (see Table 1). This result is also notable in that there is evidence that scores for ADHD decrease from childhood to (early) adulthood [58], while apparently this is not the case for CDS. Further, besides a previous study [29], it appears that this is only the second longitudinal study on CDS spanning from childhood to (early) adulthood. As such, we add to the very sparse longitudinal research on CDS. 

### 4.2. CDS Scores and Psychological Ill-Being

Based on previous studies [1,2,9,21,36,59], we assumed that higher scores for CDS at both time points (early adulthood and childhood) were associated with higher scores for depression, anxiety, stress, and insomnia at the specific time points studied, and data did confirm this assumption. As such, we replicated what has already been observed elsewhere. We also confirmed that higher scores for CDS were associated with internalizing issues such as depression, anxiety, and insomnia. More specifically, for depression, there is sufficient evidence from clinical and non-clinical samples that CDS is more strongly associated with internalizing symptoms, including depression, than ADHD-IN [9,28,29,60,61,62].

For insomnia, there is also sufficient evidence from clinical and non-clinical samples that CDS is associated with insomnia [9,11,12,21,63,64]. However, the present pattern of results expands upon previous results in that we observed an intertwined association of childhood CDS, depression, anxiety, stress, and insomnia and adulthood CDS and insomnia.

Importantly, with the first research question (Table 2), we showed that, along with higher scores for childhood CDS, dimensions of psychological ill-being both in childhood and adulthood were independently associated with higher scores for CDS in adulthood. 

The answer to the second research question is important. Childhood CDS was associated with adulthood CDS both directly and indirectly via increased scores for adult depression and stress (Figure 1 and Figure 2). We claim that this finding opens up the opportunity for psychotherapeutic interventions. First, interventions to improve CDS are scarce, mixed, and often at the level of pilot studies and open trials, ranging from the administration of ADHD-recommended stimulants to mindfulness awareness practices [1,2,6,8,65,66]. However, such sparse interventions have not yet been tested in adults with CDS. By contrast, there are sufficient standardized and evidence-based CBT and meditation/relaxation interventions to improve depression and stress [67]. As such, though highly speculative and based on the transdiagnostic CDS link [59], it is conceivable that, among adults with CDS, improving their stress-coping strategies and depression might also improve CDS scores.

Several limitations are important to note. First, the quality of the data does not allow for understanding to what extent the present pattern of results is specific to CDS and not also to ADHD in general and to ADHD-IN specifically. However, the present findings add to the mounting evidence of the strong CDS–psychological ill-being link, which was confirmed for the developmental time points of childhood and (early) adulthood. Second, one statistical approach was the conditional effects model, which is appropriate for cross-sectional and quasi-longitudinal data, though ‘genuine’ longitudinal data (e.g., [28]) would have allowed for running mediation models. Third, symptoms of CDS were solely self-assessed, while a thoroughly performed clinical interview for general psychiatric disorders [68] and specifically for CDS [69] would have allowed for a more comprehensive and critical view of the CDS scores. Fourth, one might question the quality and reliability of a quasi-longitudinal design. However, there is sufficient scientific literature focusing on a broad variety of scientific domains such as Huntington’s disease [30], female adults’ physical activity patterns [32], non-suicidal self-injury [31], adult insomnia [33,34], and post-partum depression [35], which all relied on retrospective data gathering and, thus, on quasi-longitudinal study designs. As such, we claim that the present study design is reliable. Fifth, we acknowledge the risk of recall bias due to current mood state, as was well described in the seminal work on mood-congruent memory bias [70,71,72]. However, to remember the time of childhood, we applied the imagination technique, which, in the field of CBT interventions, is very well established for insomnia [51], for relaxation in general [52], and above all for relaxation among professional athletes [53,54]. However, we also recognize that future studies should assess if and to what extent the imagination induction was successful.

## 5. Conclusions

Childhood CDS and adulthood CDS were highly associated, suggesting a stable CDS trajectory from childhood to early adulthood. Further, data suggest a strong CDS–psychological ill-being link, which, however, might offer the opportunity for depression- and stress-related psychotherapeutic interventions to improve adult CDS.

## Figures and Tables

**Table 1:** Descriptive and correlational statistical indices for scores for adolescent CDS, depression, and insomnia, and adulthood CDS, depression, and insomnia (N = 245).

	Adulthood					Childhood				
Adulthood	CDS	Depression	Anxiety	Stress	Insomnia	CDS	Depression	Anxiety	Stress	Insomnia
CDS	-	0.77 ***	0.74 ***	0.75 ***	0.70 ***	0.64 ***	0.54 ***	0.58 ***	0.58 ***	0.60 ***
Depression		-	0.83 ***	0.80 ***	0.71 ***	0.53 ***	0.56 ***	0.54 ***	0.55 ***	0.59 ***
Anxiety			-	0.78 ***	0.62 ***	0.55 ***	0.53 ***	0.60 ***	0.58 ***	0.57 ***
Stress				-	0.67 ***	0.48 ***	0.49 ***	0.46 ***	0.54 ***	0.54 ***
Insomnia					-	0.50 ***	0.52 ***	0.49 ***	0.54 ***	0.57 ***
Childhood										
CDS	-					-	0.80 ***	0.74 ***	0.77 ***	0.71 ***
Depression							-	0.84 ***	0.83 ***	0.73 ***
Anxiety								-	0.78 ***	0.68 ***
Stress									-	0.68 ***
Insomnia										-
M (SD)	10.31 (8.65)	3.34 (4.41)	2.33 (3.17)	5.12 (4.31)	14.59 (5.57)	7.50 (9.28)	2.65 (4.33)	2.15 (2.67)	4.23 (4.69)	10.56 (5.11)

Notes: CDS = cognitive disengagement syndrome. *** = *p* < 0.001.

**Table 2:** Multiple regression model to associate adult CDS with adult depression, anxiety, stress, and insomnia, and childhood CDS, depression, anxiety, stress, and insomnia.

Dimension	Variables	Coefficient	Standard Error	Coefficient β	t	*p*	R	R^2^	Durbin–Watson	VIF
Adult CDS	Intercept	−0.771	0.950	-	−0.811	0.418	0.856	0.734	1.98	
	Childhood CDS	0.322	0.054	0.345	5.976	<0.001				2.977
	Adult depression	0.550	0.124	0.280	4.437	<0.001				3.567
	Adult stress	0.580	0.117	0.289	4.975	<0.001				2.977
	Adult insomnia	0.280	0.078	0.180	3.588	<0.001				2.255
	Childhood depression	0.523	0.140	0.262	3.744	<0.001				4.374
	Childhood anxiety	0.544	0.204	0.168	2.67	0.008				3.531

The following variables did not reach statistical significance: adulthood anxiety, childhood stress, and childhood insomnia (ts < 0.9, ps > 0.405).

## Data Availability

Data might be made available under the following conditions: 1. Only an internationally recognized senior researcher can ask for the data set. 2. The scientific profile of the senior researcher is easily retrievable on the homepage of the institution. 3. The senior researcher contacts the corresponding author via her/his institutional email address (no @gmail.com or similar). 4. The senior researcher formulates clear-cut hypotheses; such hypotheses transparently describe the reasons as to why the data set should be provided. 5. The senior researcher describes credibly how the data set is securely stored on an institutional server, which is not accessible to a third party. 6. The senior researcher declares that the data set by no means is shared with a third party.
